# Major Lipids, Apolipoproteins, and Alterations of Gut Microbiota

**DOI:** 10.3390/jcm9051589

**Published:** 2020-05-23

**Authors:** Kyung Eun Yun, Jimin Kim, Mi-hyun Kim, Eunkyo Park, Hyung-Lae Kim, Yoosoo Chang, Seungho Ryu, Han-Na Kim

**Affiliations:** 1 Center for Cohort Studies, Total Healthcare Center, Kangbuk Samsung Hospital, School of Medicine, Sungkyunkwan University, Seoul 04514, Korea; ke.yun@samsung.com (K.E.Y.); jimin.kim@samsung.com (J.K.); mh0303.kim@samsung.com (M.-h.K.); yoosoo.chang@gmail.com (Y.C.); sh703.yoo@gmail.com (S.R.); 2 Department of Biochemistry, College of Medicine, Ewha Womans University, Seoul 07804, Korea; eunkyo85@gmail.com (E.P.); hyung@ewha.ac.kr (H.-L.K.); 3 Department of Occupational and Environmental Medicine, Kangbuk Samsung Hospital, School of Medicine, Sungkyunkwan University, Seoul 03181, Korea; 4 Department of Clinical Research Design and Evaluation, SAIHST, Sungkyunkwan University, Seoul 03063, Korea; 5 Medical Research Institute, Kangbuk Samsung Hospital, School of Medicine, Sungkyunkwan University, Seoul 03181, Korea

**Keywords:** gut microbiota, blood lipid, triglyceride, apolipoprotein, 16S rRNA

## Abstract

The gut microbiota has been linked to blood lipids. However, the relationship between the gut microbiome and other lipid markers like apolipoproteins A1 (apoA1) and B (apoB) as well as classical lipid markers in Asians remain unclear. Here, we examined the associations between gut microbial diversity and taxonomic compositions with both apolipoproteins and lipid markers in a large number of Korean patients. The fecal 16S rRNA gene sequencing data from 1141 subjects were analyzed and subjects were categorized into control group (G0) or abnormal group (G1) according to blood lipid measurements. The microbial diversity and several taxa of the gut microbiota were significantly associated with triglyceride, apoA1, and apoB levels, but not with total cholesterol, low-density lipoprotein cholesterol, and high-density lipoprotein cholesterol levels. The alpha diversity of the gut microbiota was inversely associated with high triglyceride level. Interestingly, G1 of apoA1 showed increased microbial richness and distinct microbial community compared with G0 of apoA1. A high abundance of Fusobacteria and low abundance of *Oscillospira* were found in the hypertriglyceridemia group. In this large-scale study, we identified associations of gut microbiota with apolipoproteins and classical lipid markers, indicating that the gut microbiota may be an important target for regulating blood lipids.

## 1. Introduction

Dyslipidemia is an important risk factor for cardiovascular disease (CVD) and refers to elevated levels of low-density lipoprotein cholesterol (LDL-C), total cholesterol (TC), and triglycerides (TG) and low levels of high-density lipoprotein cholesterol (HDL-C) [1]. Blood lipid levels are influenced by genetic [2] and environmental factors such as diet [3] and their interactions. The digestion of dietary lipid and its subsequent absorption and packaging as lipoprotein is a complex process [4]. Traditional lipid parameters as well as apolipoproteins have been identified to play a role in many mechanisms related to CVD. Apolipoprotein B (apoB) is the major apolipoprotein of the atherogenic lipoproteins, very low-density lipoprotein (VLDL), intermediate density lipoprotein (IDL), and LDL-C, while apolipoprotein A1 (apoA1) is the primary protein associated with HDL-C [5]. Nevertheless, a large proportion of the variation in blood lipid levels remains unexplained.

In recent years, the gut microbiota has emerged as an important player in both host metabolic and immunological phenotypes with important consequences for human health [6]. Gut microbiota has been reported to be associated with lipid metabolism through its role in bile acid metabolism. Gut microbiota has the ability to modulate dietary lipid composition, digestion, and absorption, potentially altering intestinal lipoprotein formation [7]. Nevertheless, there are few studies of the association between the gut microbiota and lipid markers in humans [1,8]. Most studies on lipid markers were animal studies [9], the supplementation of specific strains of bacteria [10], or human studies with a small sample size [11]. One study in humans with a sample size of 893 participants reported that alpha-diversity of gut microbiota was negatively associated with triglycerides but positively associated with HDL-C [12]. Although several microbes were associated with lipid levels, the associations between the gut microbiota and other blood lipid levels except TG have been inconsistent [1,8,12]. Furthermore, few studies have reported on the correlation between the gut microbiota and apolipoprotein levels.

A recent study showed that ethnicity is consistently associated with specific microbial taxa with a slightly higher effect size than other variables such as body mass index (BMI), and some taxa vary in abundance between ethnicities, of which the microbiota mostly were heritable and associated with genetic variation [13]. However, there have been few studies of gut microbiota and blood lipids markers in Asian populations compared with such studies in Caucasians.

In the current study, we examined cross-sectional associations between gut microbial diversity and taxonomic compositions with both classic blood lipid marker and apolipoprotein in a large number of Korean subjects (*N* = 1,141).

## 2. Methods

### 2.1. Study Subjects

Participants were recruited from the Kangbuk Samsung Health Study, which is a cohort study of Korean men and women who undergo comprehensive annual or biennial examinations at the Kangbuk Samsung Hospital Healthcare Screening Center in South Korea [14]. Over 80% of participants were employees of various industrial companies throughout the country and their spouses. In South Korea, industrial safety and health regulations require annual or biennial health screening examinations of all employees, free of charge. Traditional lipid testing is performed for all participants in these screenings, but the availability of apolipoprotein testing varies by company. Therefore, apolipoprotein data were not available for some subjects. Fecal samples were collected from 1463 participants aged 23 to 78 years who underwent a comprehensive health examination between June 2014 and September 2014 and gave informed consent for this study. Participants who met any of the exclusion criteria were excluded from the analysis. We excluded 322 subjects based on the following criteria: missing the traditional lipid levels including TC, LDL-C, HDL-C, and TG (*n* = 26); history of cancer (*n* = 52); the use of antibiotics within 6 weeks prior to enrollment (*n* = 54); the use of lipid-lowering drugs (*n* = 78) or glucose-lowering agents (*n* = 33) within the past year; and samples with less than 5000 sequences per sample (*n* = 79). A total of 1141 participants were included for the TC, LDL-C, HDL-C, and TG, and 847 participants were included for apoA1 and apoB in the final analysis.

The present study was conducted according to a protocol approved by the Institutional Review Boards of Kangbuk Samsung Hospital (2013-01-245-12). We obtained written consent from all participants after the nature and possible consequences of the studies were explained. All applicable institutional and governmental regulations concerning the ethical use of human volunteers were followed during this research. The research was carried out under the Declaration of Helsinki.

### 2.2. Data Collection and Group Definitions

Data on family history, medication use, medical history, physical activity, smoking status, alcohol consumption, and sociodemographic characteristics were collected using a self-administered, structured questionnaire. Blood pressure and anthropometric parameters were measured by trained staff during the health examination [15]. BMI was calculated as weight (kg) divided by height (m^2^). We collected blood samples after at least 10 hours of fasting. Total cholesterol and triglycerides in serum were determined with an enzymatic colorimetric assay. LDL-C and HDL-C were measured directly with a homogeneous enzymatic colorimetric assay, while apoA1 and apoB levels were measured using an immunoturbidimetric assay. The classification of blood lipid levels was based on the third report of the National Cholesterol Education Program (also called ATP III) [16]. We categorized subjects into the control group (G0) or the abnormal group (G1) according to blood lipid measurements. High total cholesterol (G1) was defined as serum total cholesterol ≥200 mg/dL, high LDL-C (G1) was defined as LDL-C ≥130 mg/dL, high TG (G1) was defined as TG ≥150 mg/dL, and low HDL-C (G1) was defined as <40 mg/dL for men and <50 mg/dL for women. For apolipoproteins, high apoB level was defined as ≥90 mg/dL [17], and low apoA1 level was defined as <120 mg/dL for men and <140 mg/dL for women [18]. The Laboratory Medicine Department at Kangbuk Samsung Hospital is accredited by the Korean Society of Laboratory Medicine and participates in annual inspections and surveys administered by the Korean Association of Quality Assurance for clinical laboratories and the College of American Pathologists Proficiency Testing.

### 2.3. DNA Extraction from Fecal Samples and 16S rRNA Gene Sequencing

Fecal samples were immediately frozen at −20 °C after defecation and stored at −70 °C within 24 h. DNA extraction from fecal samples was performed within one month of storage using the MOBio PowerSoil® DNA Isolation Kit (MO BIO Laboratories, Carlsbad, CA, USA) according to the manufacturer’s instructions. Amplification and sequencing were performed to analyze bacterial communities, as described previously [19]. Genomic DNA was amplified using fusion primers targeting the variable V3 and V4 regions of the 16S rRNA gene with indexing barcodes. Samples were pooled for sequencing on the Illumina Miseq platform (Illumina, San Diego, CA, USA) according to the manufacturer’s instructions [20]. The DADA2 [21] plugin of the QIIME2 package (version 2019.7, https://qiime2.org/) [22] was used to perform the sequence quality control, such as filtering low-quality sequences and chimeras, and to construct a feature tables of amplicon sequence variants (ASVs). The ASVs were generated by denoising with DADA2 and regarded as 100% operational taxonomic units (OTUs). For taxonomic structure analysis, taxonomy was assigned to ASVs using a pre-trained naïve Bayes classifier and the q2-feature-classifier plugin against the Greengene 99% OTUs (version 13_8) of the 16S rRNA sequence database in the QIIME2 package. Contingency-based filtering was used to filter features from a table contingent on the number of samples in which they were observed. We filtered features that were present in only one sample, based on the assumption these did not represent real biological diversity but were polymerase chain reaction (PCR) or sequencing errors such as PCR chimeras.

### 2.4. Statistical Analysis

Basic statistical analyses were conducted with RStudio (version 0.98.983, RStudio, Boston, MA, USA). Before diversity analysis, the feature table was rarefied to 5030 sequences per sample by random subsampling in QIIME2. To evaluate the alpha diversity, we calculated the number of ASVs observed in each sample, the Shannon index representing both evenness and richness, and Pielou’s evenness and Faith’s phylogenetic diversity (PD) [23]. The Mann–Whitney *U* test was used to test differences between pairwise groups. For measuring beta diversity, we used UniFrac distance [24] to estimate dissimilarity among group members by incorporating the phylogenetic distances between ASVs. The unweighted and weighted UniFrac distances were calculated to determine the presence/absence and the abundance of ASVs, respectively. Non-phylogenetic indices such as Bray–Curtis dissimilarities [25] were also used for abundance data. Pairwise permutational multivariate analysis of variance (PERMANOVA) with 999 random permutations was used to test the significance of differences between groups.

The analysis of composition of microbiome (ANCOM) [26] test was performed to determine if there were significant differences in the relative abundance of any taxa between two groups across multiple taxonomic levels. ANCOM compares the relative abundance of taxa among multiple groups by the log-ratio of the abundance of each taxon to the abundance of all the remaining taxa one at a time. To adjust for confounding variables (age, sex, and BMI), we used the ANCOM2 code shared by the author from the original ANCOM paper [26], which has the capability to deal with covariates. The final significance was expressed in the empirical distribution of W from analyses for two groups. We also conducted multivariate association with linear models (MaAsLin) [27], which has the capability to deal with covariates, and compared abundance of taxa between two groups. Linear discriminant analysis effect size (LEfSe) analysis was used to detect potential lipid-specific bacterial markers [28].

For functional inferences regarding the microbial community, we ran the Phylogenetic Investigation of Communities by Reconstruction of Unobserved States 2 (PICRUSt2) (v2.2.0-b) [29] with ASVs according to the instruction of the PICRUSt2 homepage. Phylogenetic placement in PICRUSt2 is based on the following three steps: hidden markov models (HMMER) (www.hmmer.org) to place ASVs, evolutionary placement algorithm-NG (EPA-NG) [30] to determine the best position of these placed ASVs in a reference phylogeny, and genesis applications for phylogenetic placement analysis (GAPPA) [31] to output a tree of the most likely ASV placements. This results in a phylogenetic tree containing both reference genomes and environmentally sampled organisms, which is used to predict individual gene family copy numbers for each ASV. PICRUSt2 predictions were supported by enzyme classification numbers (EC numbers, as of 21 January 2016). We generated PICRUSt2 EC gene family predictions and Metabolic Pathway Database (Metacyc) pathway abundance predictions [32]. Results were visualized in statistical analysis of taxonomic and functional profiles (STAMP) version 2.1.3 (Robert Beiko, Halifax, NS, Canada) [33] and tested using Welch’s *t*-test for two groups, good vs. poor. All predictions were adjusted for multiple testing correction (Bonferroni *q-value* <0.05).

## 3. Results

### 3.1. Subject Demographics

Table 1 shows the characteristics of the study participants. Among the total 1141 subjects, 712 (62.4%) were men and 429 (37.6%) were women. Among the overall 1141 subjects, 544 (47.7%) showed increased TC (G1), 414 (36.3%) showed increased LDL-C (G1), 162 (14.2%) showed low HDL-C (G1), 259 (22.7%) showed increased TG (G1), 202 (23.8%) showed low levels of apoA1 (G1), and 488 (57.6%) showed high levels of apoB (G1).

### 3.2. Fecal Microbial Diversity between the G0 and G1 Groups

**Figure 1:**
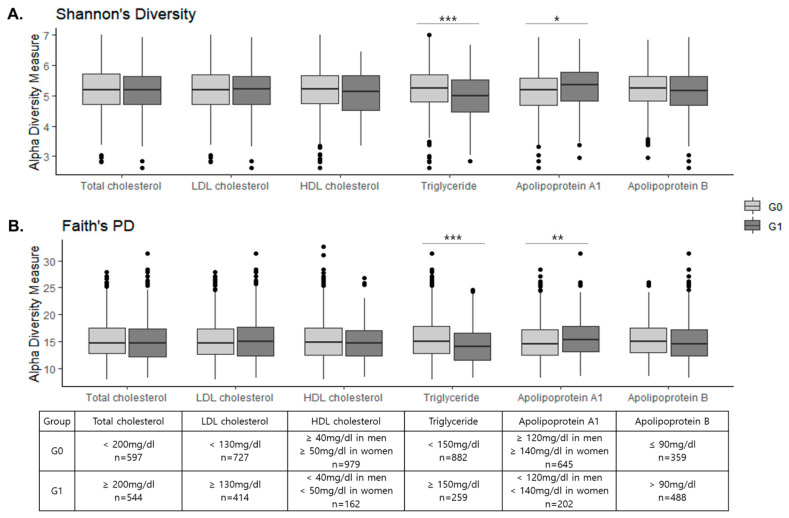
Alpha diversity and blood lipids. (**A**) Shannon’s index (*p* < 0.001 and *p* = 0.009 for triglycerides (TG) and Apo A1, respectively; Mann-Whitney *U* test), (**B**) Faith’s phylogenetic diversity (*p* < 0.001 and *p* = 0.026 for TG and Apo A1, respectively; Mann–Whitney U test) * *q* < 0.05, ** *q* < 0.01, *** *q* < 0.001 (Benjamini–Hochberg correction). The box plots indicate the interquartile range (IQR). The IQR is the 25th to 75th percentile. The median value is shown as a line within the box. Whiskers extend to the most extreme value within 1.5 × IQR. Possible outliers are indicated as dots.

The mean depth of sequences was 24,259 reads per sample and the number of features was 3431 in the 1141 subjects after contingency-based filtering of features. After rarefying the feature tables to 5030 sequences per sample, we found that the high TG group showed significantly lower richness in both phylogenetic and non-phylogenetic alpha diversity indices including observed ASVs (*p* < 0.001), Shannon’s index (*p* < 0.001), Pielou’s evenness (*p* = 0.030), and Faith’s PD (*p* < 0.001) (Figure 1 and Table S1). For apoA1, the low group (G1) showed greater diversity in Shannon’s index (*p* = 0.009) and Faith’s PD (*p* = 0.026), but there was no difference in the evenness. There was no significant difference between G0 and G1 groups in alpha diversity for other lipid measurements.

The beta diversity analysis indicates the extent of similarities and differences among microbial communities. To quantify the beta diversity, both non-phylogenetic (Bray–Curtis dissimilarity) and phylogenetic methods (Unifrac distance) were used for multiple lipid measurements (Table 2). For TG, apoA1, and apoB, we not only found significant differences between G0 and G1 in the phylogenetic Unifrac distance (*p* < 0.05, PERMANOVA), but also identified dissimilarities between them in the non-phylogenetic Bray–Curtis index (*p* < 0.05, PERMANOVA). However, due to the high sample numbers and interindividual variation, the fecal microbiota between the G0 and G1 groups could not be separated clearly by principal coordinates analysis (Figure S1), although the differences in microbial community composition were significant between the two groups in all beta diversity indices. For TC, LDL-C, and HDL-C, we could not find statistical differences in the gut microbial community composition.

### 3.3. Association of Gut Microbiota with Lipid Measurements

**Figure 2:**
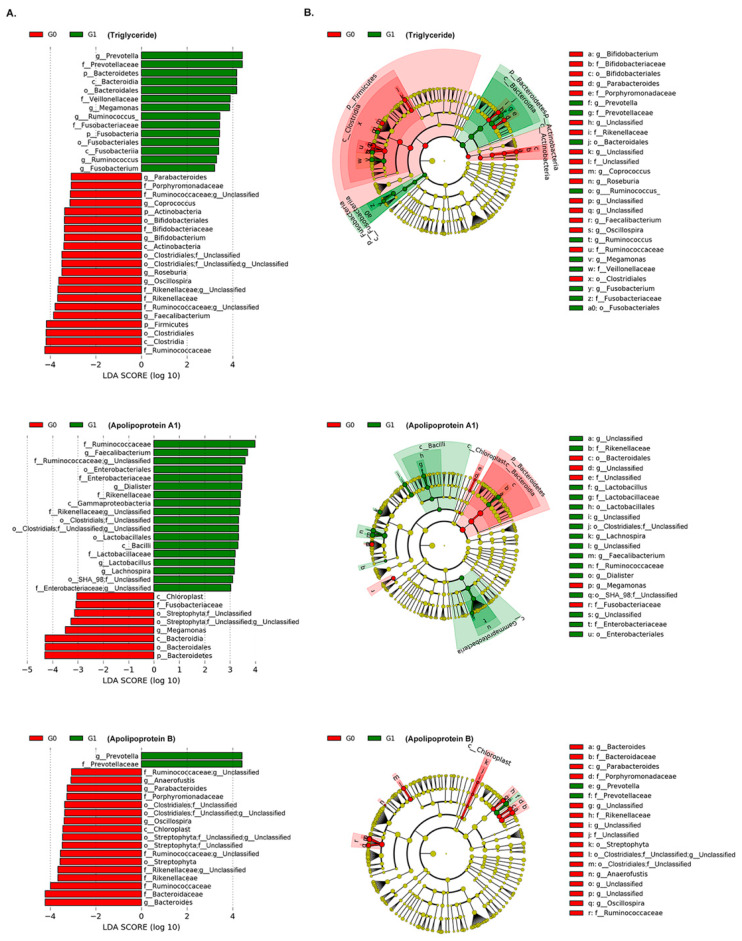
Differentially abundant bacterial taxa in fecal samples from the control (G0) and the hypertriglyceridemia group (G1). (**A**) A forest plot showing the LDA score (effect size) indicating significant differences in the bacterial taxa between the G0 (red) and G1 (green) groups for TG, ApoA1, and ApoB (LDA score > 3.0; *p* < 0.05). (**B**) Cladogram generated using the LEfSe method indicating the phylogenetic distribution of microbes associated with the G0 and the G1 groups for TG, ApoA1, and ApoB.

We investigated the associations between gut microbial composition and blood lipid markers. Based on W statistics by ANCOM, we identified 12 taxa, 10 taxa, and 6 taxa significantly associated with TG, apoA1, and apoB, respectively, from phylum to species level after adjusting for age, sex and BMI (Table 3). The TG level was positively associated with the genus *Fusobacterium* (*W* = 218), as well as their higher taxonomic levels including phylum, class, order, and family. The genera *Oscillospira* (*W* = 218) and *Anaerostipes* (*W* = 211) showed a strongly negative association with TG. The three genera *Megamonas*, *Megasphaera*, and *Acidaminococcus*, belonging to the family *Veillonellaceae* showed higher abundance in the high group (G1) than in the G0 for TG. The genus *Prevotella* showed positive associations both with TG as well as apoB in ANCOM, but these findings were not significant in MaAsLin. For apoA1, the order Lactobacillales belonging to the class Bacilli, RF39, the family *Enterobacteriaceae* belonging to the order Enterobacteriales, and Odoribacteraceae showed higher abundance in the G1 group. The genera *Odoribacter* and *Lachnospira* and the species *Bacteroides caccae* also showed significantly positive associations with ApoA1 in both ANCOM and MaAsLin. The genus *Oscillospira* (*W*=201), which was negatively associated with TG, also showed negative associations with apoB. The family *Rikenellaceae* and the genus *Clostridium* showed lower abundances in the G1 group than in the G0 group. The genus *Prevotella* and the species *copri* belonging to the family *Prevotellaceae* showed positive associations with apoB. However, we did not identify any taxa associated with TC, HDL-C, or LDL-C using true W statistics in ANCOM. The results revealed significant differences in the composition of gut microbiota mainly for TG among multiple lipid measurements. We also performed LEfSe analysis to detect potential bacterial markers that most likely explain differences between G0 and G1 groups by coupling standard tests for statistical significance with an additional test encoding biological consistency and effect relevance [28]. LEfSe analysis also confirmed that the genera *Prevotella* and *Fusobacterium* were significantly enriched in the high group (G1) for TG and the genus *Oscillospira* was enriched in the low group (G0) (LDA > 3; *p* < 0.05, Figure 2); we could not confirm the association of the genera *Acidaminococcus* and genus *Anaerostipes* with TG. For apoA1, the family *Enterobacteriaceae* and order Enterobacteriales within Gammaproteobacteria and the order Lactobacillales within the class Bacilli showed significantly higher abundance in the G1 group than in the G0 group (LDA > 3; *p* < 0.05, Figure 2). For apoB, the enrichments of the genus *Prevotella* and the species *copri* in G1 group and that of the genus *Oscillospira* in the G0 group was also confirmed in LEfSe analysis (LDA > 3; *p* < 0.05, Figure 2). Interestingly, the family *Fusobacteriaceae* showed a different direction of association in TG and apoA1 in LEfSe analysis, although the taxa showed a significantly positive association with TG only.

### 3.4. Functional Profiling Related to the Multiple Lipid Measurements

**Figure 3:**
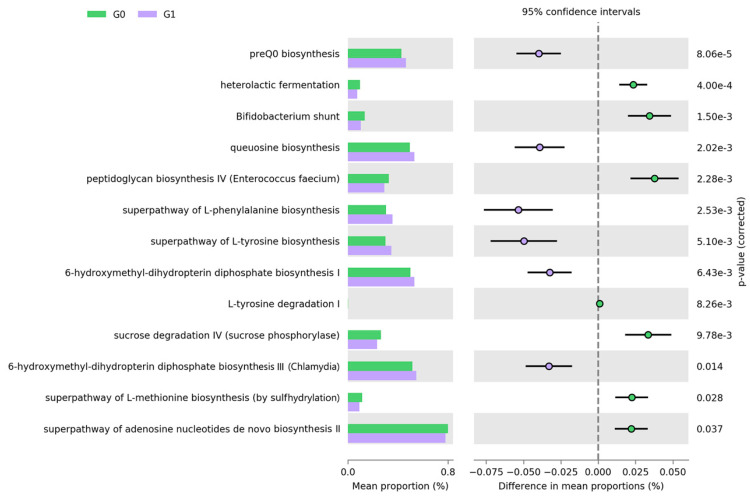
Prediction of metagenome functional content correlated with hypertriglyceridemia using Phylogenetic Investigation of Communities by Reconstruction of Unobserved States (PICRUSt2). Extended error bar plot for each pathway indicates the difference in mean proportions for each pair of groups. Two-sided Welch’s *t*-test produced a *q* < 0.05, adjusted using the Bonferroni correction.

To evaluate differences in community functional attributes, we used PICRUSt2. Among the predicted MetaCyc pathways inferred by PICRUSt2 for ASVs, we found 13 statistically significant pathways only in TG groups (Bonferroni *q*-value < 0.05) and no significant pathways at other lipid measurements (Figure 3). We found that the short-chain fatty acid (SCFA) producing-related pathways, such as heterolactic fermentation, *Bifidobacterium* shunt, and sucrose degradation IV (sucrose phosphorylase), were decreased in the G1 group compared with the G0 group for TG (*q* < 0.01). The genus *Bifidobacterium*, which showed a high LDA score in the G0 group for TG, was the top contributor of SCFA-producing pathways. The biosynthesis pathways of essential nutrients, such as preQ_0_ biosynthesis, queuosine, and 6-hydroxymethyl-dihydropterin diphosphate biosynthesis, were enriched pathways in the G1 group. Although the function of the queuine nucleoside (queuosine) has not been definitively established, its ubiquity in living organisms implies a critical biological role. Amino acid biosynthesis, such as L-phenylalanine and L-tyrosine biosynthesis, were highly detected in the G1 group.

## 4. Discussion

In this large-scale cross-sectional study, we identified associations between gut microbiota and blood apolipoproteins as well as classical lipid markers of cardiometabolic risk. The microbial diversity and several taxa of the gut microbiota were significantly associated with the blood levels of TG, apoA1 and apoB, while no association was identified for TC, LDL-C, and HDL-C levels. The alpha diversity of gut microbiota was inversely associated with hypertriglyceridemia. Interestingly, the gut microbiome showed increased richness and distinct microbial community in subjects with low levels of apoA1 compared with those in subjects with high levels of apoA1. These results suggest that the gut microbiota might affect specific aspects of lipid metabolism and distinct classes of lipoproteins and apolipoproteins [34].

TG was consistently reported to show a negative correlation with microbial diversity in previous human studies [1,8,12]. The studies also reported negative correlations for LDL-C and positive correlations for HDL-C with microbial richness. To date, however, most studies were focused on European populations. Our results, including the low alpha diversity in the high TG group in Korean subjects, were also consistent with prior studies. However, there was no significant difference in the microbial richness by LDL-C and HDL-C levels. These results might reflect ethnic differences in the effects of gut microbiota on blood lipids. Ethnicity, which reflects socio-economic, cultural, geographic, dietary, genetic diversity, and environmental factors, was shown to influence total microbiota composition [13].

*Oscillospira* spp. was inversely correlated with TG level independent of age, sex, and BMI in this study, although the genus was previously reported to be associated with leanness or lower BMI [35,36]. *Oscillospira* spp. produce butyrate, one of the main SCFAs [37], and butyrate-producing bacteria have been associated with the beneficial metabolic effects reported from fecal transplantation from lean donors to obese patients [38], while a lower capacity to ferment complex carbohydrates was found in the microbiota of patients with obesity compared with lean controls [39]. *Oscillospira* was also enriched in the feces of gallstone patients and correlated positively with secondary bile acids [40], indicating that it may contribute to the formation of these acids as well as lipid metabolism. *Prevotella* spp. showed positive correlations with TG and ApoB levels in ANCOM, although the significance disappeared in MaAsLin, while *P. copri* showed significantly positive correlations with apoB in both ANCOM and MaAsLin. The genus *Prevotella* has been associated with an obvious increase in the high-cholesterol diet group and mostly colonizes obese individuals [41]. We also found that Fusobacteria including sub-taxa showed high abundance in the hypertriglyceridemia group. Fusobacteria have been reported to be associated with inflammation [42,43], indicating the relationship between inflammation-related bacteria and lipid levels. Inflammation is always accompanied by high levels of blood lipids [44]. Our results suggest that inflammation-related bacteria might affect blood lipid levels.

ApoA1 has been recognized to exert a beneficial role in cholesterol homeostasis and immunity, and, as a consequence, to be anti-atherogenic [45]. In addition, substantial evidence has shown that apoB is a more accurate representation of atherogenic risk caused by apoB lipoproteins than that caused by LDL-C or non-HDL cholesterol [46]. To date, however, most studies related to the gut microbiome examined lipids such as cholesterol and triglycerides or lipoproteins such as LDL-C and HDL-C, while few microbiome studies have addressed apoA1 and apoB. Tall et al. reported that lipopolysaccharide (LPS) treatment caused decreased production of apoA1, which is pro-inflammatory [45], in liver [47]. In our study, we found that the family *Enterobacteriaceae*, which belongs to Gammaproteobacteria and includes Gram-negative bacteria producing LPS, showed higher relative abundance in the group (G1) with low apoA1 than in the group with high apoA1. These results suggest that apoA1 can also modulate intestinal homeostasis and microbiota composition and, notably, that an apoA1 deficiency-driven dysbiosis can contribute to inflammation or predispose to atherosclerosis development. Furthermore, the genera *Odoribacter* and *Lachnospira* showed high relative abundance in the group with low apoA1 level. In a previous study, *Odoribacter* was reported to be highly abundant in hypercholesterolemic subjects and the isobutyric acid proportion was positively associated with *Odoribacter* abundance [48]. In relation to blood lipid markers, isobutyric acid seemed to correlate positively with LDL and negatively with HDL particle size. *Odoribacter* was recently reported to have high heritability in twin studies [49] and alterations in biodiversity and abundance have been also described in other metabolism-related pathologies such as obesity, metabolic syndrome, and diabetes [50], suggesting that the dysbiosis of these bacteria in dislipidemia subjects could have a certain hereditary component, which is common to other metabolic disorders [48]. Additionally, another study reported that mice lacking apoA1 harbored an increased abundance of Lachnospiraceae [51].

We also identified predicted functional pathways that were correlated with TG levels, which implied that gut microbiota might have potential functions in influencing lipid metabolism. For example, SCFA production-related pathways, such as heterolactic fermentation, *Bifidobacterium* shunt, and sucrose degradation IV (sucrose phosphorylase), were associated with low TG level. These pathways are related to the production of acetate, one of the main SCFAs [52], and acetate was found to inhibit adipose tissue lipolysis under physiological conditions [53]. In the liver, acetate and butyrate are the major substrates for de novo lipogenesis as well as substrates for cholesterol synthesis [54]. In addition, the pathways related to queuosine biosynthesis and amino acid biosynthesis such as L-phenylalanine and L-tyrosine tended to be associated with high TG level. In a previous study, phenylalanine and tyrosine were found to be dramatically elevated in obese subjects compared with lean participants [55]. However, our results only provide inference but not a precise reflection for microbial function because we did not investigate the entire genome but only the 16S rRNA gene.

Our study had several limitations. First, this was a single center, cross-sectional retrospective study without longitudinal follow-up data. Therefore, we could not determine whether lipid changes or gut microbiome changes occurred first. Second, because there were many missing values in the nutrient data analyzed in our study, our main results did not address the effects of diet, which is a key factor in shaping gut microbiota. Nevertheless, there were no significant differences in intake of most nutrients between G0 and G1 groups when we analyzed the subgroup of participants with available diet data (Table S2 and S3). Granado-Serrano et al. reported that differences in the gut microbiome between hypercholesterolemia and normocholesterolemia subjects seemed to be intrinsically related to subject physiology rather than external factors that have been reported to alter gut microbiota composition, such as diet [48]. The authors found that although some associations were observed between carbohydrates, fat, monounsaturated fatty acids, and saturated fatty acids levels with some taxa, the intake of these nutrients was similar in the two groups. The potential influence of the quality as well as quantity of nutrients on the differences observed in gut microbial composition between G0 and G1 groups warrants further investigation. Third, the associations observed between microbial taxa and lipid levels were best at the genus level, at least based on currently used 16S methods, although we reported them to the species level. Furthermore, 16S rRNA gene sequencing provides little information relevant to bacterial genes. Our understanding of the strains associated with blood lipid markers, their genes and functions, and metabolic pathways will be expanded through whole metagenomic sequencing in the future. Nevertheless, this study is the first report of the association of gut microbiota with apolipoprotein levels as well as blood lipid levels in Asia. A growing body of literature supports the role of the gut microbiota in lipid metabolism and dyslipidemia, but there have been relatively few Asian-based studies of gut microbiota and blood lipid levels. More studies in Asian subjects are needed in the future.

## 5. Conclusions

Our study presents associations of the gut microbiota with blood apolipoproteins and classical lipid markers. These results highlight the microbial relevance to future clinical outcomes through blood lipid regulation. Based on the potential mechanisms of the identified microbial factors, further studies in animal models and future prospective studies are needed to explain the therapeutic implications. The gut microbiome may serve as an important target in developing preventive or therapeutic strategies for dyslipidemia.

## Figures and Tables

**Table 1:** Study participant characteristics.

	Overall	Male	Female	*p* Value ^a^
No. of subjects	1141	712	429	
Age (years), mean (SD)	45.2 (8.7)	45.7 (8.7)	44.4 (8.7)	0.015
BMI (kg/m^2^)	23.5 (3.1)	24.6 (2.8)	21.8 (2.8)	<0.001
Current smoker (%)	17.2	26.9	1.17	<0.001
Alcohol intake (%) ^b^	38.1	55.2	9.7	<0.001
Vigorous exercise (%) ^c^	14.5	14.7	14.2	0.474
SBP (mmHg), mean (SD)	109.4 (13.5)	114.2(12.2)	101.5 (11.8)	<0.001
DBP (mmHg), mean (SD)	71.0 (10.1)	74.3 (9.3)	65.5 (9.0)	<0.001
Fasting glucose (mg/dL), mean (SD)	94.7 (14.3)	97.3 (16.5)	90.4 (7.7)	<0.001
Total cholesterol (mg/dL), mean (SD)	198.7 (32.4)	202.2 (32.6)	192.9 (31.2)	<0.001
G0: <200 mg/dL (*N*)	597	348	249	
G1: ≥200 mg/dL (*N*)	544	364	180	
LDL cholesterol (mg/dL), mean (SD)	120.7 (29.4)	125.5 (28.7)	112.8 (28.9)	<0.001
G0: <130 mg/dL (*N*)	727	402	325	
G1: ≥130 mg/dL (*N*)	414	310	104	
HDL cholesterol (mg/dL), mean (SD)	57.0 (14.4)	53.1 (13.0)	63.4 (14.4)	<0.001
G0: ≥40 mg/dL in men ≥50 mg/dL in women	979	622	357	
G1: <40 mg/dL in men <50 mg/dL in women	162	90	72	
Triglyceride (mg/dL), mean (SD)	120.4 (75.3)	137.4 (83.5)	92.1 (47.6)	<0.001
G0: <150 mg/dL (N)	882	492	390	
G1: ≥150 mg/dL (N)	259	220	39	
ApoA1 (mg/dL), mean (SD)	143.7 (22.4)	140.5 (22.8)	149.1 (20.7)	<0.001
G0: ≥120 mg/dL in men ≥140 mg/dL in women	645	443	202	
G1: <120 mg/dL in men <140 mg/dL in women	202	91	111	
ApoB (mg/dL), mean (SD)	95.5 (23.1)	101.3 (21.9)	85.6 (21.6)	<0.001
G0: <90 mg/dL (N)	359	166	193	
G1: ≥90 mg/dL (N)	488	368	120	
hsCRP (mg/dL), p25/p50/p75	0.02/0.04/0.08	0.03/0.05/0.09	0.02/0.03/0.06	<0.001

ApoA1, apolipoprotein A1; ApoB, apolipoprotein B; BMI, body mass index; DBP, diastolic blood pressure; G0, group 0; G1, group 1; HDL, high-density lipoprotein; hsCRP, high sensitivity C-reactive protein; LDL, low-density lipoprotein; SBP, systolic blood pressure; SD, standard deviation. ^a^ *p* value for difference between male and female by *t*-test for continuous variables and the chi-squared test for categorical variables. ^b^ ≥10 g of ethanol per day. ^c^ more than three times per week.

**Table 2:** Statistical significance between G0 and G1 groups using distance matrices for the beta-diversity.

Group	Total Cholesterol (*n* = 1141)	LDL Cholesterol (*n* = 1141)	HDL Cholesterol (*n* = 1141)	Triglyceride (*n* = 1141)	Apolipoprotein A1 (*n* = 847)	Apolipoprotein B (*n* = 847)
G0	<200 mg/dL *n* = 597	<130 mg/dL *n* = 727	≥40 mg/dL in men ≥50 mg/dL in women *n* = 979	<150 mg/dL *n* = 882	≥120 mg/dL in men ≥140 mg/dL in women *n* = 645	<90 mg/dL *n* = 359
G1	≥200 mg/dL *n* = 544	≥130 mg/dL *n* = 414	<40 mg/dL in men <50 mg/dL in women *n* = 162	≥150 mg/dL *n* = 259	<120 mg/dL in men <140 mg/dL in women *n* = 202	≥90 mg/dL *n* = 488
	*p*-value ^a^	*p*-value ^a^	*p*-value ^a^	*p*-value ^a^	*p*-value ^a^	*p*-value ^a^
Unweighted UniFrac distance	0.199	0.089	0.395	0.001 **	0.009 **	0.005 **
Weighted UniFrac distance	0.152	0.057	0.610	0.001 **	0.009 **	0.005 **
Bray–Curtis_dissimilarity	0.753	0.260	0.432	0.001 **	0.012 **	0.001 **

^a^ the *p*-values were calculated using pairwise permutational multivariate analysis of variance (PERMANOVA) with 999 permutations. ** *p*-value < 0.01.

**Table 3:** Significant taxa profiles of gut microbiota related with blood lipids and apolipoproteins.

		W ^a^ (Coefficients ^b^)		
Taxa level ^a^	Taxonomic Assignment	Triglyceride G0 vs. G1	Apo A1 G0 vs. G1	Apo B G0 vs. G1
Phylum	p__Fusobacteria	12 (0.015 **)		
Class	p__Fusobacteria; c__Fusobacteriia	27 (0.015 **)		
	p__Firmicutes; c__Bacilli		23 (0.010 **)	
Order	p__Fusobacteria; c__Fusobacteriia; o__Fusobacteriales	45 (0.015 **)		
	p__Proteobacteria; c__Gammaproteobacteria; o__Enterobacteriales		41 (0.014 *)	
	p__Firmicutes; c__Bacilli; o__Lactobacillales		40 (0.008 *)	
	p__Tenericutes; c__Mollicutes; o__RF39		40 (0.008 **)	
Family	p__Fusobacteria; c__Fusobacteriia; o__Fusobacteriales; f__*Fusobacteriaceae*	83 (0.015 **)		
	p__Bacteroidetes; c__Bacteroidia; o__Bacteroidales; f__*Prevotellaceae*	81 (0.024)		84 (0.033)
	p__Proteobacteria; c__Gammaproteobacteria; o__Enterobacteriales; f__*Enterobacteriaceae*		71 (0.014 *)	
	p__Bacteroidetes; c__Bacteroidia; o__Bacteroidales; f__*Odoribacteraceae*		68 (0.011 **)	
	p__Bacteroidetes; c__Bacteroidia; o__Bacteroidales; f__*Rikenellaceae*			69 (−0.002)
Genus	p__Firmicutes; c__Clostridia; o__Clostridiales; f__Ruminococcaceae; g__*Oscillospira*	223 (−0.022 **)		201 (−0.006)
	p__Fusobacteria; c__Fusobacteriia; o__Fusobacteriales; f__Fusobacteriaceae; g__*Fusobacterium*	218 (0.011 *)		
	p__Firmicutes; c__Clostridia; o__Clostridiales; f__Veillonellaceae; g__*Megamonas*	217 (0.005)		
	p__Firmicutes; c__Clostridia; o__Clostridiales; f__Veillonellaceae; g__*Megasphaera*	217 (0.004)		
	p__Firmicutes; c__Clostridia; o__Clostridiales; f__Veillonellaceae; g__*Acidaminococcus*	216 (0.004 *)		
	p__Bacteroidetes; c__Bacteroidia; o__Bacteroidales; f__Prevotellaceae; g__*Prevotella*	215 (0.024)		226 (0.033)
	p__Firmicutes; c__Clostridia; o__Clostridiales; f__Lachnospiraceae; g__*Anaerostipes*	211 (−0.010 **)		
	p__Bacteroidetes; c__Bacteroidia; o__Bacteroidales; f__Odoribacteraceae; g__*Odoribacter*		196 (0.010 **)	
	p__Firmicutes; c__Clostridia; o__Clostridiales; f__Lachnospiraceae; g__*Lachnospira*		186 (0.015 **)	
	p__Firmicutes; c__Bacilli; o__Lactobacillales; f__Lactobacillaceae; g__*Lactobacillus*		182 (0.003)	
	p__Firmicutes; c__Clostridia; o__Clostridiales; f__Clostridiaceae; g__*Clostridium*			197 (−0.002)
Species	p__Bacteroidetes; c__Bacteroidia; o__Bacteroidales; f__Bacteroidaceae; g__*Bacteroides*;s__*caccae*		310 (0.012 **)	
	p__Bacteroidetes; c__Bacteroidia; o__Bacteroidales; f__Prevotellaceae; g__*Prevotella*;s__*copri*			339 (0.046 *)

p_ = phylum; c_ = class; o_ = order; f_ = family; g_ = genus; s_ = species. The number of phylum: 13, The number of class: 28, The number of order: 46, The number of family: 85, The number of genera: 227, The number of species: 340. ^a^ W = X for taxon k, then H_0k_ is rejected X times. For W statistics, taxa-wise multiple correction was applied with adjusting for age, sex, and BMI. ^b^ the coefficients from the generalized linear model using MaAsLin on pairwise testing between two groups with adjusting for age, sex, and BMI. * *p* < 0.05, ** *p* < 0.01.

## Supplementary Materials

The following are available online at https://www.mdpi.com/2077-0383/9/5/1589/s1. Table S1: Associations between gut microbial diversity and blood lipid markers, Table S2: Nutritional statistics according to blood lipid markers, Table S3: Correlations between the gut microbiota in Table 3 and apoA1 after adjusting for age, sex, BMI, and total fat intake, Figure S1: PCoA analysis of beta-diversity indices.
