# Supplementary material for: Major Lipids, Apolipoproteins, and Alterations of Gut Microbiota
Source: J Clin Med. 2020 May 23;9(5):1589. doi: 10.3390/jcm9051589 (PMC7290464; doi:10.3390/jcm9051589)
Supplement: Supplementary file 1 [file jcm-09-01589-s001.pdf]

Supplementary Table S1. Associations between gut microbial diversity and blood lipid markers.

Group	Total cholesterol (n = 1,141)	LDL cholesterol (n = 1,141)	HDL cholesterol (n = 1,141)	Triglyceride (n = 1,141)	Apolipoprotein A1 (n = 847)	Apolipoprotein B (n = 847)
G0	< 200mg/dl<br>n=597	< 130mg/dl<br>n = 727	≥ 40mg/dl in men<br>≥ 50mg/dl in women<br>n = 979	< 150mg/dl<br>n = 882	≥ 120mg/dl in men<br>≥ 140mg/dl in women<br>n=645	< 90mg/dl<br>n = 359
G1	≥ 200mg/dl<br>n = 544	≥ 130mg/dl<br>n = 414	< 40mg/dl in men<br>< 50mg/dl in women<br>n = 162	≥ 150mg/dl<br>n = 259	< 120mg/dl in men<br>< 140mg/dl in women<br>n = 202	≥ 90mg/dl<br>n = 488
α-diversity index	*p*-value[a]	*p*-value[a]	*p*-value[a]	*p*-value[a]	*p*-value[a]	*p*-value[a]
Observed_ASVs	0.320	0.968	0.490	<0.001	0.056	0.149
Shannon index	0.621	0.921	0.212	<0.001	0.009	0.247
Pielou's evenness	0.862	0.861	0.282	0.030	0.151	0.722

Faith's PD	0.263	0.998	0.263	<0.001	0.026	0.115

[a] The *p*-values were calculated using the Mann-Whitney U test.

Supplementary Fig S1. PCoA analysis of beta-diversity indices. PCoA plots based on unweighted UniFrac distance (A, B, and C), weighted UniFrac distances (D, E, and F), and Bray-Curtis dissimilarity (G, H, and I) of gut microbiome labeled by triglyceride (TG), apolipoprotein A1 (apoA1), and apolipoprotein B (apoB). Symbols with different colors represent group 0 (G0; control; blue dots) and group 1 (G1; high TG, low apoA1, and high apoB; orange dots)

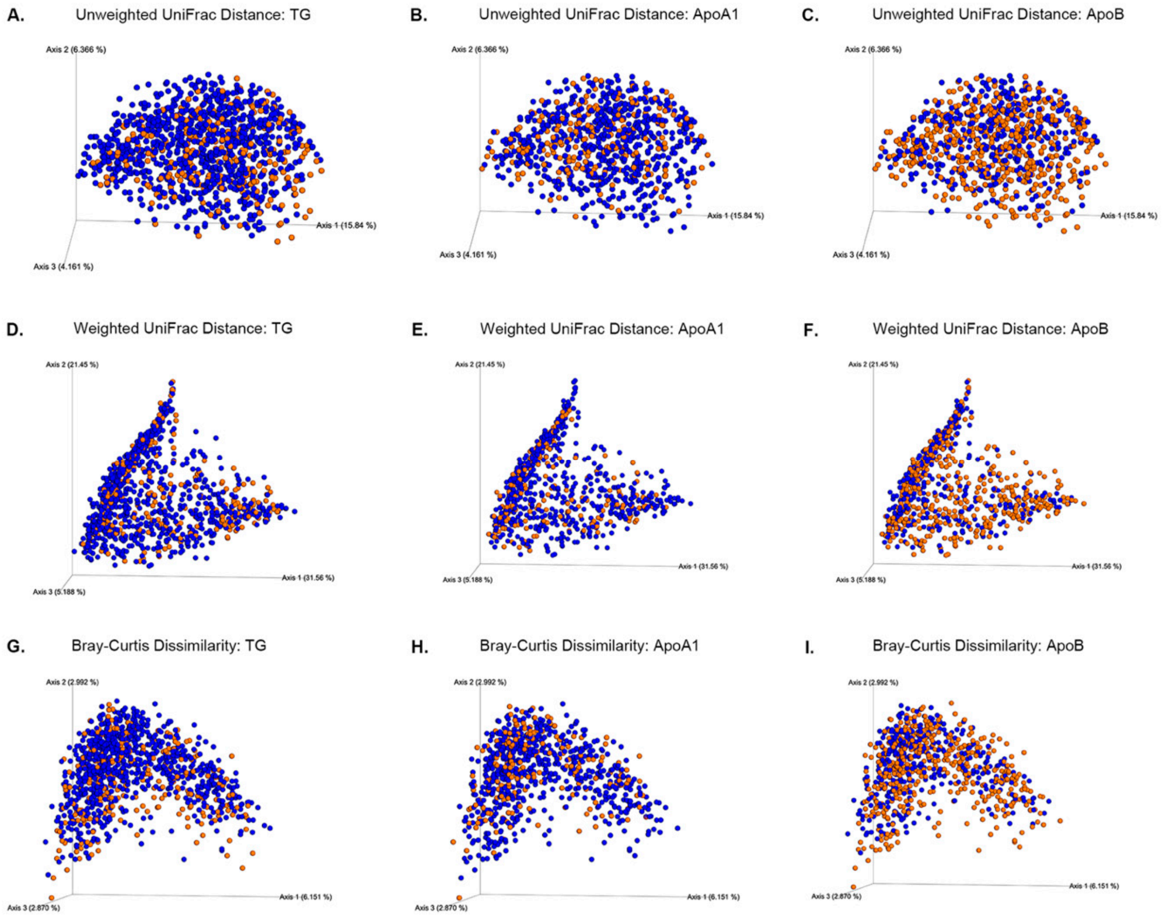

Supplementary Table S2. Nutritional statistics according to blood lipid markers.

		Total cholesterol (n=836)	LDL cholesterol (n=836)	HDL cholesterol (n=836)	Triglyceride (n=836)	Apolipoprotein A1 (n=623)	Apolipoprotein B (n=623)
Group 0 (G0)		<200mg/dl<br>n=435	<130mg/dl<br>n=528	≥40mg/dl in men<br>≥50mg/dl in women<br>n=718	<150mg/dl<br>n=652	≥120mg/dl in men<br>≥140mg/dl in women<br>n=471	<90mg/dl<br>n=255
Group 1 (G1)		≥200mg/dl<br>n=401	≥130mg/dl<br>n=308	<40mg/dl in men<br><50mg/dl in women<br>n=118	≥150mg/dl<br>n=184	<120mg/dl in men<br><140mg/dl in women<br>n=152	≥90mg/dl<br>n=368
Total energy, kcal/day, mean (SD)	G0,	1399.6(624.1)	1391.1(613.2)	1416.6(618.1)	1389.1(621.2)	1413.3(650.1)	1395.5(656.8)
	G1	1408.7(616.3)	1426.0(632.0)	1327.1(628.5)	1456.7(614.6)	1349.6(621.0)	1399.4(630.9)
	p-value	0.831	0.433	0.146	0.191	0.286	0.941
	G0	47.3(24.5)	47.7(24.4)	48.7(24.4)	47.6(24.4)	48.9(26.1)	47.2(24.8)

Total protein, g/day, mean (SD)	G1	48.9(24.2)	48.7(24.5)	44.4(23.8)	49.8(24.3)	45.2(22.9)	48.6(25.8)
	p-value	0.348	0.572	0.078	0.293	0.118	0.493
Total fat, g/day, mean (SD)	G0	27.3(19.0)	28.0(19.4)	28.5(18.8)	27.8(19.0)	29.3(20.2)	28.3(20.9)
	G1	28.7(18.3)	27.8(17.3)	24.2(17.4)	28.3(17.2)	25.5(17.0)	28.4(18.5)
	p-value	0.278	0.911	0.017*	0.747	0.024*	0.943
Total carbohydrate, g/day, mean (SD)	G0	237.3(107.6)	233.4(105.1)	237.4(105.1)	233.5(105.6)	234.8(108.0)	234.6(112.5)
	G1	235.1(104.1)	241.1(107.8)	229.1(112.3)	246.2(107.9)	231.3(109.0)	233.5(105.2)
	p-value	0.766	0.314	0.432	0.152	0.728	0.903
Fiber, g/day, mean (SD)	G0	3.7 (2.3)	3.7 (2.2)	3.6 (2.1)	3.7 (2.2)	3.6 (2.2)	3.7 (2.3)
	G1	3.67 (2.1)	3.7 (2.1)	3.7 (2.1)	3.7 (2.1)	3.7 (2.3)	3.6 (2.2)
	p-value	0.797	0.989	0.875	0.974	0.603	0.734

Dietary consumption was assessed using a 103-item self-administered food frequency questionnaire (FFQ) designed for use in Korea (Ahn, Y., Kwon, E., Shim, J.E., et al., Validation and reproducibility of food frequency questionnaire for Korean genome epidemiologic study. Eur J Clin Nutr **2007**, 61, 1435-1441.

* $p < 0.05$

Supplementary Table S3. Correlations between the gut microbiota in Table 3 and apoA1 after adjusting for age, sex, BMI, and total fat intake.

		Coefficients		
		ApoA1 (G0 vs. G1)	ApoA1 (G0 vs. G1)	Total fat intake
Taxa level	Taxonomic assignment	Adjusting for age, sex, and BMI	Adjusting for age, sex, BMI, and total fat intake	Coefficients (p-value)
Class	p__Firmicutes;c__Bacilli	0.010**	0.010**	-0.000 (0.955)
Order	p__Proteobacteria;c__Gammaproteobacteria;o__Enterobacteriales	0.014*	0.014*	-0.000 (0.485)
	p__Firmicutes;c__Bacilli;o__Lactobacillales	0.008*	0.008*	0.000 (0.979)
	p__Tenericutes;c__Mollicutes;o__RF39	0.008**	0.008**	0.000 (0.963)
Family	p__Proteobacteria;c__Gammaproteobacteria;o__Enterobacteriales;f__*Enterobacteriaceae*	0.014*	0.014*	-0.000 (0.500)
	p__Bacteroidetes;c__Bacteroidia;o__Bacteroidales;f__*Odoribacteraceae*	0.011**	0.011**	0.000 (0.422)
Genus	p__Bacteroidetes;c__Bacteroidia;o__Bacteroidales;f__Odoribacteraceae;g__*Odoribacter*	0.010**	0.001**	0.000 (0.269)
	p__Firmicutes;c__Clostridia;o__Clostridiales;f__Lachnospiraceae;g__*Lachnospira*	0.015**	0.015**	-0.000 (0.367)
	p__Firmicutes;c__Bacilli;o__Lactobacillales;f__Lactobacillaceae;g__*Lactobacillus*	0.003	0.003	0.000 (0.531)
Species	p__Bacteroidetes;c__Bacteroidia;o__Bacteroidales;f__Bacteroidaceae;g__*Bacteroides*;s__ *caccae*	0.012**	0.012**	-0.000 (0.731)

*$p < 0.05$, **$p < 0.01$
